# Assessing the Usability of an Automated Continuous Temperature Monitoring Device (iThermonitor) in Pediatric Patients: Non-Randomized Pilot Study

**DOI:** 10.2196/10804

**Published:** 2018-12-21

**Authors:** Sujay S Kakarmath, Emily de Redon, Amanda Jayne Centi, Ramya Palacholla, Joseph Kvedar, Kamal Jethwani, Stephen Agboola

**Affiliations:** 1 Harvard Medical School Boston, MA United States; 2 Partners Connected Health Boston, MA United States; 3 Massachusetts General Hospital Boston, MA United States

**Keywords:** connected health, continuous monitoring, mobile phone, pediatric, temperature

## Abstract

**Background:**

Fever is an important vital sign and often the first one to be assessed in a sick child. In acutely ill children, caregivers are expected to monitor a child’s body temperature at home after an initial medical consult. Fever literacy of many caregivers is known to be poor, leading to fever phobia. In children with a serious illness, the responsibility of periodically monitoring temperature can add substantially to the already stressful experience of caring for a sick child.

**Objective:**

The objective of this pilot study was to assess the feasibility of using the iThermonitor, an automated temperature measurement device, for continuous temperature monitoring in postoperative and postchemotherapy pediatric patients.

**Methods:**

We recruited 25 patient-caregiver dyads from the Pediatric Surgery Department at the Massachusetts General Hospital (MGH) and the Pediatric Cancer Centers at the MGH and the Dana Farber Cancer Institute. Enrolled dyads were asked to use the iThermonitor device for continuous temperature monitoring over a 2-week period. Surveys were administered to caregivers at enrollment and at study closeout. Caregivers were also asked to complete a daily event-monitoring log. The Generalized Anxiety Disorder-7 item questionnaire was also used to assess caregiver anxiety at enrollment and closeout.

**Results:**

Overall, 19 participant dyads completed the study. All 19 caregivers reported to have viewed temperature data on the study-provided iPad tablet at least once per day, and more than a third caregivers did so six or more times per day. Of all participants, 74% (14/19) reported experiencing an out-of-range temperature alert at least once during the study. Majority of caregivers reported that it was easy to learn how to use the device and that they felt confident about monitoring their child’s temperature with it. Only 21% (4/9) of caregivers reported concurrently using a device other than the iThermonitor to monitor their child’s temperature during the study. Continuous temperature monitoring was not associated with an increase in caregiver anxiety.

**Conclusions:**

The study results reveal that the iThermonitor is a highly feasible and easy-to-use device for continuous temperature monitoring in pediatric oncology and surgery patients.

**Trial Registration:**

ClinicalTrials.gov NCT02410252; https://clinicaltrials.gov/ct2/show/NCT02410252 (Archived by WebCite at http://www.webcitation.org/73LnO7hel)

## Introduction

Critical pediatric illness can be a major source of stress for parents. Fever is a common symptom in postoperative pediatric patients as well as in those with neutropenia [1,2]. Even though most fevers within 48 hours of a surgery are benign and self-limiting, fever can be a sign of underlying complication and parents are expected to be vigilant [1,3]. Furthermore, pediatric patients with cancer undergoing chemotherapy are predisposed to infectious complications because of neutropenia induced by myelosuppressive therapy and require caregivers to be watchful for even longer periods of time [3,4]. In both cases, fever is the first clinical sign of infection, and early detection is essential to evaluate the risk for further complications and death [4,5]. Therefore, continued monitoring of body temperature may be helpful in detecting any sudden changes in body temperature that may be related to a significant cause of illness in children [4].

Furthermore, monitoring a child periodically for fever can add to the already stressful experience of taking care of a sick child, leading to fever phobia, a well-documented phenomenon in parents [6]. Moreover, previous studies show that parental knowledge about normal body temperature and the temperature that indicates fever is often poor, and few parents can accurately take temperature measurements [7-9]. Even in parents who do not belong to any of these groups, the process of monitoring fever periodically can be significantly disruptive to daily routine and necessitate interruption of sleep. Thus, automated and continuous fever monitoring for children can overcome several problems described above.

The iThermonitor is a continuous temperature-monitoring device that can transmit temperature data to a mobile phone app paired with the device. However, the availability of this novel technology may not necessarily translate into its adoption due to parental concerns and low receptivity toward new technology. The primary aim of this study was to evaluate the feasibility of using a US Food and Drug Administration-approved automated device for continuous temperature monitoring in postoperative and postchemotherapy pediatric patients. We also evaluated the usability, satisfaction, and engagement of caregivers with the device. Finally, we assessed whether continuous temperature monitoring inadvertently increased caregiver anxiety.

## Methods

### Study Objective

We conducted a pilot study to evaluate the feasibility of using the iThermonitor for continuous temperature monitoring in postoperative and postchemotherapy pediatric patients.

### Recruitment and Study Procedures

Formal enrollment in the study occurred during an in-person enrollment visit scheduled with patients and caregivers. At the enrollment visit, after explaining study details and procedures, pediatric participants and their caregivers were given sufficient time to review the consent form and encouraged to ask questions. Caregivers consented to the study on behalf of pediatric participants and were asked to complete the enrollment questionnaire. Pediatric participants aged 10-17 years were also required to confirm their willingness to participate in the study by signing an assent form. An informed consent form was signed and collected prior to the study. The enrollment questionnaire was administered after obtaining informed consent, which contained questions on demographic information, caregiver technology use, and Generalized Anxiety Disorder-7 questionnaire (GAD-7; Multimedia Appendices 1 and 2). Each day, pediatric participants were asked to wear the device while caregivers were asked to complete an “event-monitoring log” every day over the study period of 14 days (Multimedia Appendix 3). Participants were given instructions to attach the iThermonitor to the skin using a hydrogel dressing that can be changed as needed. Temperature data collected by the iThermonitor were automatically uploaded to a paired receiver (an iPad Mini tablet computer) within a range of 5 m for cloud storage. The provided iPad Mini was preloaded with the iThermonitor app that was used to pair the receiver with the iThermonitor device.

Temperature data were then downloaded and stored in the Partners Healthcare network files. Data files were available to only the Partners Institutional Review Board-approved study staff at Partners Connected Health. If participants required hospital admission, they were asked to stop using the device during their hospital stay. If such a stay resulted in <50% of data being collected, participants were administratively dropped from the study. All participants were asked to continue to receive medical treatment and adhere to other management protocols as recommended by their physicians. After 14 days of use, participants were either scheduled for a closeout visit to return the devices and complete the closeout questionnaire (Multimedia Appendices 2 and 4, respectively) or were sent an electronic questionnaire via Research Electronic Data Capture (an electronic study data capture system) along with shipping material to return their study devices.

### Intervention

**Figure 1:**
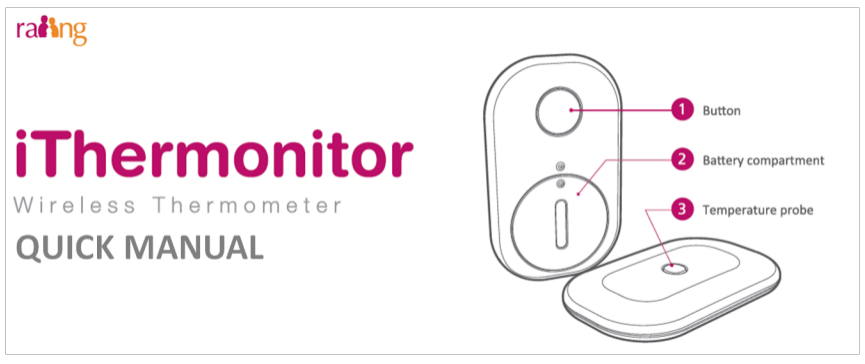
iThermonitor device.

The iThermonitor (Figure 1) is a US Food and Drug Administration class II device that continuously captures body temperature and automatically delivers the data wirelessly (via Bluetooth or Wi-Fi) to mobile devices or for cloud storage. In addition, it generates and delivers out-of-range temperature alerts on a mobile app for caregivers or providers, allowing them to remotely monitor their child’s temperature (Multimedia Appendix 5).

### Data Collection

Feasibility of using the iThermonitor was the primary outcome of interest. Success as a feasible continuous temperature- monitoring tool was defined *a priori* as “80% of the participants viewing the temperature data on the device for at least 80% of the study period.” This was assessed in two ways:

- Participant responses from the “event-monitoring log.”
- Participant responses to the checklist administered as part of the closeout questionnaire (See below).

Please indicate Yes or No for each column every day during the study in response to the following two questions:

The iThermonitor stayed on the body for most of the day?

I was able to view the temperature data on the iPad mini?

Secondary outcomes were assessed using a closeout survey designed by study investigators to obtain caregiver feedback about the following: (1) Frequency of receiving out-of-range temperature alerts; (2) Usability of the device; (3) Acceptability of the device; and (4) Caregiver satisfaction in using the iThermonitor. These surveys were administered as part of the closeout questionnaire. Finally, GAD-7 was also administered as part of the enrollment and closeout surveys to assess change in caregiver anxiety levels [10].

### Statistical Analysis

We used descriptive statistics to characterize the study sample and survey responses. GAD-7 scores were coded as a categorical variable as follows: mild anxiety (total score 0-5) and moderate or severe anxiety (total score 6-15) [10]. The proportion of participants with mild and moderate or severe anxiety at enrollment and closeout was compared using Cochran’s Q test. All analyses were conducted using STATA (StataCorp LLC, College Station TX 77845, USA) version 14.2 with an alpha of .05 set *a priori.* Because this was an exploratory study with descriptive statistics, a complete case analysis approach was adopted for this study.

### Sample Size

Because this was a pilot study, we did not conduct formal power calculations for sample size estimations. Previous usability studies recommend a sample size of 20 users, which will identify at least 95% of usability problems [11]. We assumed a 20% loss to follow-up rate and arrived at a sample size of 25 patient-caregiver dyads.

## Results

### Participant Recruitment

**Figure 2:**
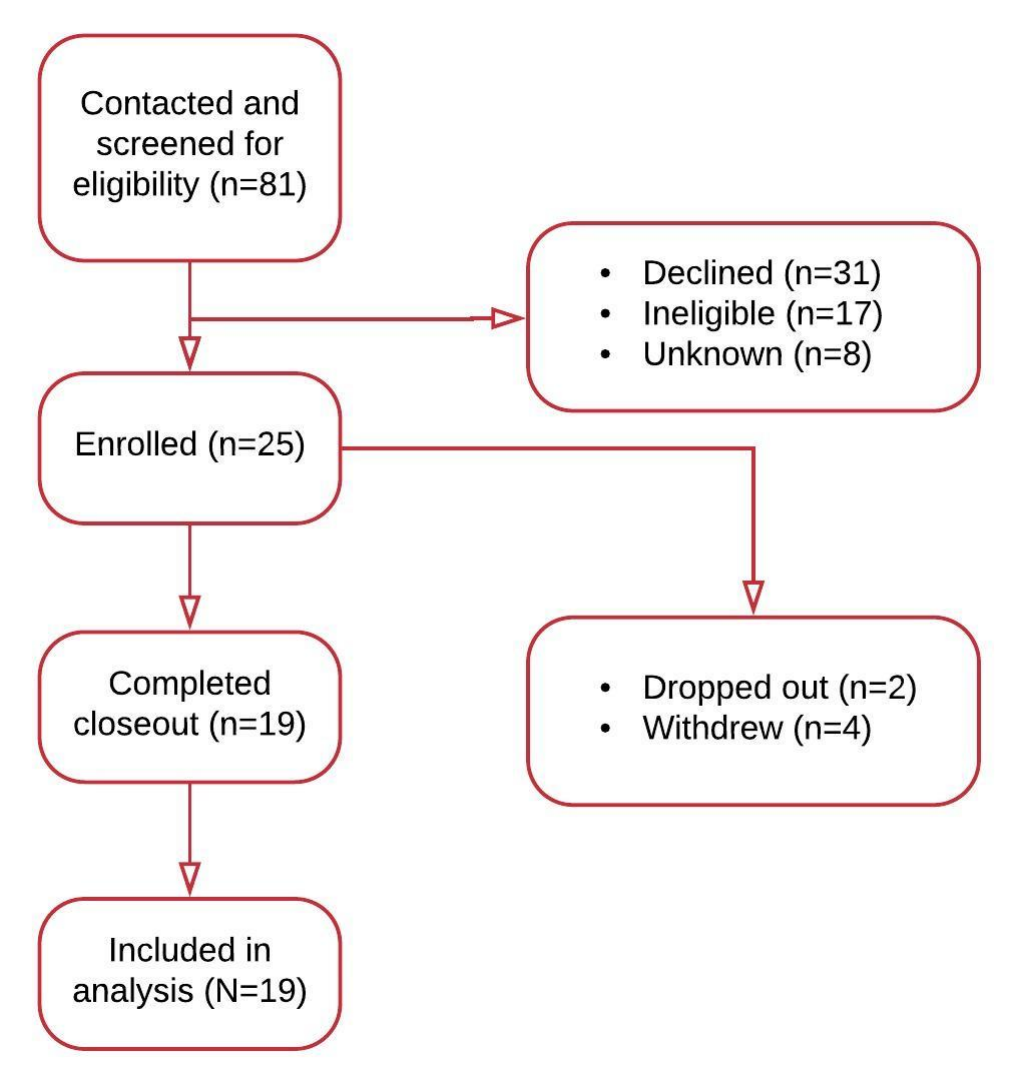
Participant enrollment flowchart.

We recruited a total of 25 patient-caregiver dyads. The first study participant was enrolled on April 24, 2015, from the MGH Department of Pediatric Oncology, and subsequently, a total of 17 participants were enrolled from this department over a period of >18 months. The first participant from the MGH Department of Pediatric Surgery site was recruited on December 23, 2016, and a total of 8 participants were enrolled, with enrollment completion on February 2, 2017. Figure 2 provides details of participant selection.

### Participant Characteristics

The mean age of participants was 8 (SD 5) years (Table 1). Of the 25 enrolled participants, 4 withdrew consent during the study and 2 were administratively dropped out. Overall, 19 participants completed the study and were included in this analysis. Of all, 94% (17/19) participants identified themselves as white, and 3 out of 4 participants were male (Table 1). The mean age of caregivers was 41 (range, 28-54) years. In addition, 1 in 2 caregivers were employed, 1 in 3 were homemakers, and the rest were unemployed. Furthermore, 63% (12/19) of study participants were pediatric oncology patients, with hematological malignancy being the most common diagnosis (Table 1). Surgical procedures varied widely among the 8 participants, with hernia repair being the only reoccurring procedure.

**Table 1:** Participant characteristics (N=19).

Characteristics			Value
Male, n (%)			14 (74)
Age of participants in years, mean (SD)			9 (6)
Age of caregivers in years, mean (SD)			41 (8)
**Race, n (%)**			
	White		17 (94)
	Asian		1 (6)
**Employment status of caregivers, n (%)**			
	Employed full-time		5 (26)
	Employed part-time		3 (16)
	Homemaker		6 (32)
	Unemployed		5 (26)
**Baseline conditions, n (%)**			
	**Malignancies**		12 (63)
		Hematologic	9 (47)
		Intracranial tumor	1 (5)
		Rhabdomyosarcoma	1 (5)
		Osteosarcoma	1 (5)
	**Surgical procedures**		7 (37)
		Hernia repair	2 (11)
		Circumcision	1 (5)
		Laparoscopy	1 (5)
		Orchiopexy	1 (5)
		Foreign body removal	1 (5)
		Unknown	1 (5)

### Attitudes Toward Technology

Most caregivers reported favorable attitudes toward technology. All caregivers reported owning smartphones and using them to access the internet, send or receive emails and short message service text messages, and share pictures. However, only two-thirds of caregivers reported using smartphones or other technology to track weight, diet, or exercise, and only one-third reported using any technology to track health.

### Feasibility

**Figure 3:**
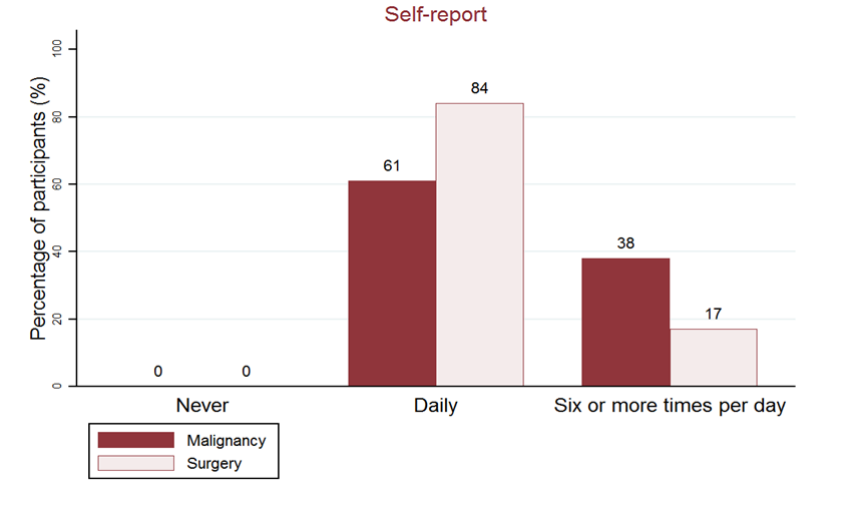
Frequency of viewing temperature on iPad.

In response to the single-item question, all caregivers indicated viewing temperature data on the iPad at least once every day (Figure 3). Majority (5/6, 84%) of caregivers of pediatric surgery patients reported viewing temperature data at least once daily. In comparison, most caregivers of pediatric oncology patients reported viewing temperature data for six or more times per day. However, only 37% (7/19) participants completed the daily event-monitoring log for 12 out of the 14 days, that is, for >80% of the study duration. Furthermore, 100% (19/19) of these participants reported that they viewed temperature data for each of the 12 days.

### Out-of-Range Temperature Alerts

In this study, 74% (14/19) of caregivers reported receiving an out-of-range temperature alert at least once during the study period. Of these, 64% (9/14) of caregivers reported receiving an alert ≥3 times.

### Usability, Acceptability, and Satisfaction

All caregivers reported that it was easy to learn to use the iThermonitor. While 84% (16/19) of caregivers reported feeling comfortable using the device to monitor their child’s temperature, 79% (15/19) reported that they could easily monitor their child’s temperature with it.

Furthermore, 74% (14/19) of caregivers reported finding the mobile app very useful in monitoring temperature. However, only 53% (10/19) found the out-of-range feature useful. While 74% (14/19) of caregivers reported feeling more confident about monitoring their child’s temperature using the device, 79% (15/19) reported that they would recommend it to a friend or a family member. Only 21% (4/19) of caregivers reported that they used another device to monitor the child’s temperature during the study.

None of the caregivers reported experiencing problems with the primary function of the device, that is, measurement of temperature. Some caregivers (n=3) had concerns about the minor differences in the temperature reported by the device compared with another thermometer used by them. Most problems reported by caregivers were related to the nonclinical features of the device. For example, few caregivers (n=3) found wireless range of the device to be limited; some (n=5) found it difficult to sync the mobile app with the device. Some caregivers (n=4) occasionally experienced problems with attaching the tape to the child’s body or keeping it in place, and others (n=3) found low battery life of the iThermonitor to be bothersome.

### Caregiver Anxiety

While 16% (3/19) of participants had mild anxiety, 21% (4/19) had moderate or severe anxiety at enrollment. At closeout, 11% (2/19) participants had mild anxiety and 5% (1/19) had moderate or severe anxiety. However, this difference in proportions was not statistically significant (*P*=.29).

## Discussion

### Principal Findings

We conducted a pilot study to evaluate the feasibility of using the iThermonitor as a home-based, continuous temperature- monitoring tool in postoperative and postchemotherapy pediatric patients. The iThermonitor may be a feasible tool to replace conventional temperature monitoring in pediatric patients. Caregivers reported that it was easy to use and increased their confidence in monitoring the child’s temperature. Our findings demonstrated that caregivers are willing to engage with continuous temperature-monitoring devices, without experiencing an increase in anxiety. This finding is important considering the well-documented phenomenon of fever phobia [5].

We used two methods for measurement of feasibility: self- reported response in the closeout questionnaire and daily event-monitoring log. The estimates of feasibility of using the device obtained using the self-reported response (16/19, 84%) were substantially higher than those obtained through the daily event-monitoring log (7/19, 37%). The lower estimate of feasibility from the event-monitoring log is likely an artifact of the added burden on study participants to complete one log for each day of participation in the study. In contrast, the burden of participation in the one-time, self-reported response in the closeout questionnaire was much lower. Therefore, despite the possibility of response bias involved with the self-reported questionnaire, it is likely to be a better estimator of feasibility in this study.

Fever is one of the first and most common complications in pediatric surgical patients [12]. Discharge instructions for caregivers often require them to monitor body temperature and take definitive action if it crosses a threshold [9]. However, fever literacy in caregivers has been reported to be low in previous studies [13]. A systematic review of the literature concluded that parental knowledge about body temperature monitoring is poor [7]. Parents have been reported to base their fever management practices on inaccurate temperature readings [7]. Pediatric illnesses are associated with substantial stress experienced by caregivers, and some studies have also reported that parents worry about failing to recognize a serious problem in their acutely sick child [14]. The ability of the caregiver to stay at home with the child and monitor vital signs such as temperature can vary by socioeconomic factors such as education, literacy, income, and marital status [15]. The stress resulting from these factors is only compounded in caregivers of pediatric patients who have a serious illness that requires surgery or prolonged medical treatment [16,17].

Digital health technologies are particularly well suited to eliminate human error from relatively simple tasks in home-based caregiving such as body temperature measurement [18]. These technologies also offer an easy, safe, and comfortable method to monitor body temperature in pediatric patients [5]. In addition, digital health technologies such as the iThermonitor provide a unique opportunity to caregivers to access important data (temperature readings) through the convenience of a phone or tablet computer, thereby eliminating the burden of constant temperature monitoring by caregivers. Furthermore, the out-of-range temperature alerts feature may help reduce caregivers’ stress by bringing their attention to any unwanted changes in body temperature.

### Limitations

One major limitation of this study is the lack of a control group that used a regular device for temperature measurement such as a digital thermometer without a companion app or automated temperature measurement features. Therefore, we are unable to ascertain the benefit of these features relative to a simple digital thermometer. However, this feasibility pilot study can set the stage for a larger trial to compare clinical and other patient-reported outcomes in patients. Second, we evaluated caregiver engagement through self-reported data at closeout, and thus, these results may be subject to recall bias. Third, parental perception of the novelty of the device may have biased them to provide more favorable responses to the usability and satisfaction assessment. Finally, our study sample represents a relatively narrow selection of pediatric illnesses. Therefore, our findings may not hold true in other pediatric illnesses and in the general population. Hence, a larger sample size is required to evaluate the long-term impact of such continuous monitoring devices.

### Conclusion

Overall, the iThermonitor is an easy-to-use device that is highly feasible for continuous monitoring of temperature in pediatric oncology and surgery patients. Most parents quickly developed sufficient confidence in the device to not use any other temperature-monitoring device during the study. Although findings from this pilot study have limited generalizability, a device such as the iThermonitor may have the potential to reduce caregiver stress resulting from taking care of a sick child around the clock. Finally, it may also improve caregivers’ knowledge on temperature fluctuations and help them better monitor their children.

## Appendix

Multimedia Appendix 1 iThermonitor Enrollment Questionnaire.

Multimedia Appendix 2 GAD-7.

Multimedia Appendix 3 iThermonitor Daily Event Monitoring Log.

Multimedia Appendix 4 iThermonitor Closeout Questionnaire.

Multimedia Appendix 5 User Manual.

## Abbreviations

DFCI: Dana Farber Cancer Institute
GAD-7: Generalized Anxiety Disorder Questionnaire
MGH: Massachusetts General Hospital

## References

[1] Yeung R S, Buck J, Filler R (1982). THe significance of fever following operations in children. J Pediatr Surg.

[2] Celkan T, Koç BŞ (2015). Approach to the patient with neutropenia in childhood. Turk Pediatri Ars.

[3] te Poele Esther M, Tissing Wim J E, Kamps Willem A, de Bont Eveline SJM (2009). Risk assessment in fever and neutropenia in children with cancer: What did we learn?. Crit Rev Oncol Hematol.

[4] Toma A, Fenaux P, Dreyfus F, Cordonnier C (2012). Infections in myelodysplastic syndromes. Haematologica.

[5] Vijarnsorn C, Winijkul G, Laohaprasitiporn D, Chungsomprasong P, Chanthong P, Durongpisitkul K, Soonswang J, Nana A, Subtaweesin T, Sriyoschati S, Pooliam J (2012). Postoperative fever and major infections after pediatric cardiac surgery. J Med Assoc Thai.

[6] El-Radhi AS, Barry W (2006). Thermometry in paediatric practice. Arch Dis Child.

[7] Crocetti M, Moghbeli N, Serwint J (2001). Fever phobia revisited: have parental misconceptions about fever changed in 20 years?. Pediatrics.

[8] Walsh A, Edwards H (2006). Management of childhood fever by parents: literature review. J Adv Nurs.

[9] Porter R S, Wenger F G (2000). Diagnosis and treatment of pediatric fever by caretakers. J Emerg Med.

[10] Spitzer RL, Kroenke K, Williams JBW, Löwe B (2006). A brief measure for assessing generalized anxiety disorder: the GAD-7. Arch Intern Med.

[11] Faulkner L (2003). Beyond the five-user assumption: benefits of increased sample sizes in usability testing. Behav Res Methods Instrum Comput.

[12] Torreggiani S, Filocamo Giovanni, Esposito Susanna (2016). Recurrent Fever in Children. Int J Mol Sci.

[13] Wallenstein MB, Schroeder AR, Hole MK, Ryan C, Fijalkowski N, Alvarez E, Carmichael SL (2013). Fever literacy and fever phobia. Clin Pediatr (Phila).

[14] Kai J (1996). What worries parents when their preschool children are acutely ill, and why: a qualitative study. BMJ.

[15] Shudy Marysia, de Almeida Mary Lihinie, Ly Susan, Landon Christopher, Groft Stephen, Jenkins Tammara L, Nicholson Carol E (2006). Impact of pediatric critical illness and injury on families: a systematic literature review. Pediatrics.

[16] Patiño-Fernández Anna Maria, Pai Ahna L H, Alderfer Melissa, Hwang Wei-Ting, Reilly Anne, Kazak Anne E (2008). Acute stress in parents of children newly diagnosed with cancer. Pediatr Blood Cancer.

[17] Commodari E (2010). Children staying in hospital: a research on psychological stress of caregivers. Ital J Pediatr.

[18] Monsma J, Richerson Julia, Sloand Elizabeth (2015). Empowering parents for evidence-based fever management: An integrative review. J Am Assoc Nurse Pract.

