# Supplementary material for: Assessing the Usability of an Automated Continuous Temperature Monitoring Device (iThermonitor) in Pediatric Patients: Non-Randomized Pilot Study
Source: JMIR Pediatr Parent. 2018 Dec 21;1(2):e10804. doi: 10.2196/10804 (PMC6716441; doi:10.2196/10804)
Supplement: Multimedia Appendix 1 [file pediatrics_v1i2e10804_app1.pdf]

Supplementary Material 1

iThermonitor Enrollment Questionnaire **Study ID: ________________**

Date: ________________

There are three sections in this form – A, B and C. Section A contains questions about your child. Sections B and C contains questions for the caregiver.

Section A: Child-specific information

Please answer the following questions about your child.

1. What is your child's age? _____(Years) or _____(Months)

2. What is your child's gender?
(Circle the number of your answer.)

1 Male

2 Female

3. Is your child Hispanic or Latino?
(Circle the number of your answer.)

1 Yes

2 No

4. Which one or more of the following would you say is your child's race?
(Circle the number of your answers, more than one may apply.)

1 American Indian or Alaska Native

2 Asian

3 Black or African American

4 Native Hawaiian or other Pacific Islander

5 White

6 Other, please specify: ______________________________

Section B: Caregiver-specific information

Please answer the following questions about yourself.

1. What is your age? ___________ *(Write in number of years.)*

2. What is your gender?
(Circle the number of your answer.)

1 Male

2 Female

3. What is your marital status?
(Circle the number of your answer.)

1 Married

2 Living with partner

3 Divorced or separated

4 Widowed

5 Single, never been married

5. What is the highest grade at school or years in college that you have completed?
(Circle the number of your answer.)

1 1st – 8th grade

2 9th – 11th grade

3 12th grade, completed high school, or GED

4 1 to 3 years of college

5 4 or more years of college

6. Are you Hispanic or Latino?
(Circle the number of your answer.)

1 Yes

2 No

7. Which one or more of the following would you say is your race?
(Circle the number of your answers, more than one may apply.)

1 American Indian or Alaska Native

2 Asian

3 Black or African American

4 Native Hawaiian or other Pacific Islander

5 White

6 Other, please specify: ______________________________

8. What is your current employment status?
(Circle the number of your answer.)

1 Employed full-time (includes self-employment)

2 Employed part-time (includes self-employment)

3 Unemployed

4 Homemaker

5 Student

6 Retired

7 Disabled

8 Other, please specify: _____________________________

Section C: Technology Ownership and Use

1. Do you ever go online to access the Internet or World Wide Web, or to send and receive email? *(Circle the number of your answer.)*

1 Yes

2 No [skip to question 4]

2. When you use the Internet, do you access it through…

(*For each item, please circle the number for your answer.*

*You may answer "YES" to more than one question.***)**

	**Yes**	**No**
a. A regular dial-up telephone line.	1	2
b. Broadband such as DSL, cable or FiOS.	1	2
c. A cellular network (e.g., smartphone, 3G/4G)	1	2
d. A wireless network (Wi-Fi)	1	2

3. Have you ever used the Internet to do any of the following things?

(*For each item, please circle the number for your answer.***)**

	**Yes**	**No**
a. Send or receive email.	1	2
b. Send or receive instant messages or chat online.	1	2
c. Upload pictures to share with others.	1	2
e. Look for health or medical information online.	1	2
f. Track weight, diet or exercise routine.	1	2
g. Track any other health indicators like blood pressure, sleep patterns, headaches, etc.	1	2

h. Check your bank account balance or do any online banking.	1	2
i. Use a social networking service like Facebook or MySpace.	1	2

4. Do you have any of the following devices?

(*For each item, please circle the number for your answer.***)**

	**Yes**	**No**
a. A landline telephone.	1	2
b. A desktop computer.	1	2
c. A laptop computer or netbook.	1	2
d. A tablet computer like an iPad.	1	2

5. Have you ever used your cell phone or smartphone to do any of the following things?

(*For each item, please circle the number for your answer.***)**

	**Yes**	**No**
a. Send or receive email.	1	2
b. Send or receive text messages.	1	2
c. Take a picture to share with others.	1	2
d. Access the Internet.	1	2
e. Look for health or medical information online.	1	2

f. Track weight, diet or exercise routine.	1	2
g. Track any other health indicators like blood pressure, sleep patterns, headaches, etc.	1	2
h. Check your bank account balance or do any online banking.	1	2
i. Use a social networking service like Facebook or MySpace.	1	2

6. **How much do you agree or disagree with <u>each</u> of the following statements?**
(*For each item, please circle the number for your answer.*)

	**Strongly agree**	**Somewhat agree**	**Somewhat disagree**	**Strongly disagree**
a. I am willing to try new technology.	1	2	3	4
b. I plan to make greater use of technology in the future to manage my health.	1	2	3	4
c. I generally feel confident using new technology.	1	2	3	4
d. I generally feel confident that I can use new technology to manage my medical condition.	1	2	3	4
e. I worry about security issues of sending health information by the Internet.	1	2	3	4
f. It is easy for me to follow instructions and set up new technology.	1	2	3	4

g. I have no difficulty setting up computers or Internet modems.	1	2	3	4
h. Learning how to use new technology is easy for me.	1	2	3	4
i. New technology can be useful in keeping me healthy.	1	2	3	4
j. I find using new technology to be a waste of time.	1	2	3	4
