# Supplementary material for: Assessing the Usability of an Automated Continuous Temperature Monitoring Device (iThermonitor) in Pediatric Patients: Non-Randomized Pilot Study
Source: JMIR Pediatr Parent. 2018 Dec 21;1(2):e10804. doi: 10.2196/10804 (PMC6716441; doi:10.2196/10804)
Supplement: Multimedia Appendix 2 [file pediatrics_v1i2e10804_app2.pdf]

Supplementary Material 2

Subject ID: _________________

Visit: _________________

Date: _________________

GAD-7

**Over the last 2 weeks, how often have you been bothered by the following problems?** *(Use "✔" to indicate your answer)*	**Not at all**	**Several days**	**More than half the days**	**Nearly every day**
**1.** Feeling nervous, anxious or on edge	0	1	2	3
**2.** Not being able to stop or control worrying	0	1	2	3
**3.** Worrying too much about different things	0	1	2	3
**4.** Trouble relaxing	0	1	2	3
**5.** Being so restless that it is hard to sit still	0	1	2	3
**6.** Becoming easily annoyed or irritable	0	1	2	3
**7.** Feeling afraid as if something awful might happen	0	1	2	3

(*For office coding: Total Score T____* = ____ + ____ + ____)
