# Supplementary material for: Assessing the Usability of an Automated Continuous Temperature Monitoring Device (iThermonitor) in Pediatric Patients: Non-Randomized Pilot Study
Source: JMIR Pediatr Parent. 2018 Dec 21;1(2):e10804. doi: 10.2196/10804 (PMC6716441; doi:10.2196/10804)
Supplement: Multimedia Appendix 3 [file pediatrics_v1i2e10804_app3.pdf]

Supplementary Material 3

Subject ID: __________   Date of Enrollment: _________

iThermonitor Daily Event Monitoring Log

Please indicate **Yes** or **No** for each column everyday during the study.

**Day**	**The iThermonitor stayed on the body for most of the day**		**I was able to view the temperature data on the iPad mini**	
	**Yes**	**No**	**Yes**	**No**
**1**				
**2**				
**3**				
**4**				
**5**				
**6**				
**7**				
**8**				
**9**				
**10**				
**11**				
**12**				
**13**				
**14**				
