# Supplementary material for: Assessing the Usability of an Automated Continuous Temperature Monitoring Device (iThermonitor) in Pediatric Patients: Non-Randomized Pilot Study
Source: JMIR Pediatr Parent. 2018 Dec 21;1(2):e10804. doi: 10.2196/10804 (PMC6716441; doi:10.2196/10804)
Supplement: Multimedia Appendix 4 [file pediatrics_v1i2e10804_app4.pdf]

Supplementary Material 4

iThermonitor Closeout Questionnaire **Study ID:** ________________

Date: ________________

_**Thank you participating in the iThermonitor study. Please answer the following questions about your experience using the device to monitor your child's temperature.**

1. **The following are statements about your experience using the iThermonitor. Please rate how much the following statements concerning the iThermonitor are true for you.** (*Please circle the number of your answer)***:**

		**Definitely NOT true**	**A little bit true**	**Mostly true**	**Definitely true**
1)	Overall, I am satisfied with how easy it was to use the device	1	2	3	4
2)	It was easy learning to use the device	1	2	3	4
3)	I felt comfortable using the device	1	2	3	4
4)	I was able to easily monitor my child's temperature by using this device	1	2	3	4
5)	I feel more confident monitoring my child's temperature by using the device	1	2	3	4
6)	The device was helpful in starting discussions about my child's health with my doctor	1	2	3	4
7)	Using the device makes me feel more connected to my care team	1	2	3	4
8)	I found the mobile application very useful in monitoring my child's temperature	1	2	3	4

9)	I found the out-of-range temperature alert function very useful when my child's temperature was out of range	1	2	3	4
10)	I would recommend iThermonitor to a friend or family member	1	2	3	4
11)	The iThermonitor stayed on my child's body for most days during the study.	Yes		No	
12)	I was able to view my child's temperature data on the provided iPad mini for most days during the study.	Yes		No	

2. **Did you view your child's temperature on the iThermonitor app?**
(Please circle <u>one</u> of these options)
a. Yes
b. No
c. Prefer not to answer

3. **Did you use another device other than the provided iPad mini to monitor your child's temperature? (**Please circle <u>one</u> of these options)
a. Yes
b. No
c. Prefer not to answer

4. **How often did you view the measurements?** *(Please circle* ***<u>one</u>*** *of these options)*

a. Hourly
b. 2-hourly
c. 4-hourly
d. Daily
e. 2-3 times per week
f. Once a week
g. never

h. other: _________________

5. Was viewing measurements on the app useful in being able to better manage your child's health? *(Please circle **one** of these options)*

a. Yes
b. No
c. Prefer not to answer

6. Did viewing measurements on the app help you become more interested in your child's health? *(Please circle **one** of these options)*

a. Yes
b. No
c. Prefer not to answer

7. Did you discuss the iThermonitor device with others?

*(Please circle **one** of these options)*

a. Yes
b. No
c. Prefer not to answer

8. Did you discuss the iThermonitor device with your doctor?

*(Please circle **one** of these options)*

a. Yes
b. No
c. Prefer not to answer

9. If you were given the choice would you like to continue using the iThermonitor device to monitor your child's health?

*(Please circle **one** of these options)*

a. Yes
b. No
c. Prefer not to answer

10. If the iThermonitor were for sale in a store, would you buy it?

(Please circle all that apply)

a. Yes, for my child.

b. No

c. Prefer not to answer

d. Yes, for someone else (please tell us who): ___________________________

11. How much do you like the components of the iThermonitor system below? *(Please circle the number of your answer)*

	Not at all	A little bit	I like it	Very much
a) Continuous temperature monitoring	0	1	2	3
b) Viewing measurements on the app	0	1	2	3
c) Alerts when measurements are out of range	0	1	2	3
d) Other (please specify): __________________________________ ____________________	0	1	2	3

12. Did you experience problems using the iThermonitor?

*(Please circle **one** of these options)*

a. Yes

b. No*

c. Prefer not to answer *

***[If No** or “**Prefer not to answer”**, skip to question 14]*

13. What problems did you experience?

*(Please circle **all that apply**)*

a. Problems keeping the device on my child’s body

b. Problems linking the device with my smartphones

c. Problems with the iThermonitor battery

d. Problems setting out-of-range temperature alerts

e. Other problems: ___________________________

14. Please explain in detail the nature of the problem(s) you experienced as marked above:

____

____

____

____

____

15. What else would you want added to the iThermonitor system to help to you better monitor your child’s health?

____

____

____

____

____

16. Did the iThermonitor alert you when your child’s temperature was of out-of-range during the study period?

a) Yes

b) No

c) Prefer not to answer

[If *****No** or “*****Prefer not to answer”*****, skip to question 17]***

17. How many times did the iThermonitor detect out-of-range temperatures? (If multiple times, please indicate number)

a) Once
b) Twice
c) 3 times
d) Other: ____times

18. We understand that this questionnaire may not fully capture your experiences participating in this study. Would you be interested in speaking with a research staff about your experiences in this research study?

a) Yes
b) No

Your answers are important to us. Please take a moment to check that you have answered all of the questions. Thank you for taking the time to complete this questionnaire!
