# Supplementary figures and images for: Assessing the Usability of an Automated Continuous Temperature Monitoring Device (iThermonitor) in Pediatric Patients: Non-Randomized Pilot Study
Source: JMIR Pediatr Parent. 2018 Dec 21;1(2):e10804. doi: 10.2196/10804 (PMC6716441; doi:10.2196/10804)

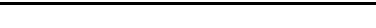

Supplement: Multimedia Appendix 5 [file pediatrics_v1i2e10804_app5.pdf]
